# mtDNA deletion m.8753_16566 with < 10 % heteroplasmy in muscle and isolated complex-V dysfunction misinterpreted as chronic fatigue syndrome over 21-years

**DOI:** 10.1016/j.clinsp.2025.100604

**Published:** 2025-03-06

**Authors:** Josef Finsterer, Ana C. Fiorini, Fulvio A. Scorza, Carla A. Scorza

**Affiliations:** a Neurology and Neurophysiology Center, Vienna, Austria; b Programa de Estudos Pós-Graduado em Fonoaudiologia, Pontifícia Universidade Católica de São Paulo (PUC-SP), São Paulo, SP, Brazil; c Departamento de Fonoaudiologia, Escola Paulista de Medicina/Universidade Federal de São Paulo (EPM/UNIFESP), São Paulo, SP, Brazil; d Escola Paulista de Medicina/Universidade Federal de São Paulo (UNIFESP/EPM), São Paulo, SP, Brazil

Chronic Fatigue Syndrome (CFS), also known as Myalgic Encephalomyelitis (ME), is a common diagnosis in patients who complain of chronic fatigue and exercise intolerance even with little physical activity.^1^ CFS is currently defined as extreme fatigue lasting at least six months and symptoms that worsen with physical or mental activity but do not fully improve at rest.^1^^,^^2^ The cause of ME/CFS is not known, but it is thought to be triggered by a combination of factors. As there is no biomarker to confirm the diagnosis, it can only be made by ruling out other health problems with similar symptoms.^2^ Treatment of the condition focuses on alleviating the symptoms.

Mitochondrial Disorders (MIDs) are a group of genotypically and phenotypically heterogeneous genetic diseases that are due to a disruption of reproduction, fusion and fission, energy production, other mitochondrial metabolic processes, membrane function, and signaling in the mitochondria.^1^ They may be due to mutations in the mitochondrial DNA (mtDNA) or in genes located in the nuclear DNA. The prevalence of MIDs in the USA is estimated at 1 in 4000 people.^3^ Single mtDNA deletions lead to mitochondrial deletion syndromes that manifest as chronic progressive external ophthalmoplegia, Pearson syndrome, Kearns-Sayre Syndrome (KSS), or non-syndromic non-specific disease.^4^ The diagnosis is made on the basis of characteristic clinical features and the detection of an mtDNA deletion of 1.1 to 10 kb in molecular genetic tests of mtDNA from blood, buccal cells, urine (children), or skeletal muscle tissue (adults).^4^ The treatment of MIDs is based on symptomatic measures.

Although fatigue and exercise intolerance are common features of MID, an adult patient with MID who has an mtDNA deletion in low heteroplasmy and has been misdiagnosed as CFS for years has not yet been reported.

The patient is a 52-year-old woman who was diagnosed with myalgic ME/CFS at the age of 25 after an infection with parvovirus B19 and has not been the same since. For the past twenty-one years, she has suffered from chronic fatigue, exercise intolerance, and post-exertional malaise and has been mostly housebound and often bedbound (up to 22 h per day). Functionally, she tipped over into an anaerobic metabolism when talking, walking, or eating. Since the age of 34, she had recurrent stroke-like symptoms, although she had never been examined by MRI of the brain for the presence of a stroke-like lesion. She had no external ophthalmoplegia, but whenever she was more fatigued, her left eyelid would droop, or she developed problems with bowel motility. Her family history was negative for metabolic disorders, especially MID. droop, or she developed problems with bowel motility. Her family history was negative for metabolic disorders, especially MID.

A muscle biopsy at the age of 39 years revealed an increased lipid content and a predominance of type II muscle fibers, which was considered a non-specific finding. An endo-myocardial biopsy for suspected viral myocarditis and an endothelial natural killer cell function test were inconclusive. VO2 max tests showed a drastic reduction in aerobic capacity. An mtDNA analysis from muscle at 46 years of age revealed the m.8753_16,566 deletion with <10 % heteroplasmy. The deletion was larger than in patients with KSS but encompassed the entire ATP6 gene associated with complex-V of the respiratory chain. An abnormal complex-V band was detected in the MitoFIND assay, indicating that the mutation was a germline mtDNA deletion and not an erroneous or fictitious finding. Native polyacryl gel electrophoresis showed incomplete assembly of complex V of the respiratory chain. The function of complex-I to IV was normal. Exome sequencing using NextSeq 550 revealed no pathogenic or likely pathogenic mutation in the nuclear exome, indicating that there was no other mutational alternative of equal significance to the mtDNA deletion. Extensive testing for multisystem disease was negative. Mitochondrial cocktails were ineffective, but intravenous immunoglobulins had a transient positive effect.

The patient presented is interesting in several respects. Firstly, the m.8753_16566del variant has not previously been described as a cause of MID. Second, the mtDNA deletion caused isolated complex-V dysfunction. Third, the mtDNA deletion manifested phenotypically despite a heteroplasmy rate of <10 % in muscle. Fourth, MID remained undetected for 21-years. Fifth, the mtDNA deletion manifested only in skeletal muscle. Sixth, the mtDNA variant first became symptomatic after a parvovirus infection. An isolated complex-V insufficiency was previously described at least in lymphocytes of ME/CFS patients.^5^ The reason why the mtDNA deletion had effects on biochemical function, complex-V protein structure and mitochondrial membrane functions despite low heteroplasmy in muscle remains unknown, but it can be speculated that heteroplasmy rates were higher in tissues other than muscle or that the low heteroplasmy was sufficient to impair mitochondrial functions due to the large deletion size. Isolated complex-V insufficiency can manifest not only as fatigue but also as severe, multisystemic MID.^6^ In one child, the m.8561C>T variant in ATP6/ATP8 manifested with early-onset ataxia, psychomotor delay and microcephaly.^6^ One argument in favor of parvovirus infection as a trigger of chronic fatigue could be the fact that infection of A549 cells with the HH6 virus led to fragmentation of the mitochondria and induction of 1-carbon metabolism, dUTPase and thymidylate synthase, while superoxide dismutase-2 and proteins required for mitochondrial oxidation of fatty acids, amino acids and glucose metabolism, including pyruvate dehydrogenase, were strongly inhibited.^7^

In summary, this case demonstrates that mtDNA deletions with low heteroplasmy rates can manifest phenotypically as chronic fatigue, exercise intolerance and post-exertional malaise, mimicking CFS. Patients diagnosed with CFS should undergo a comprehensive diagnostic workup to avoid overlooking the underlying cause, which may be amenable to treatment. Physicians should consider a small heteroplasmic mtDNA deletion as a cause of CFS.

## Data access statement

All data are available from the corresponding author.

## Ethics statement

Not applicable.

## Compliance with ethics guidelines

This article is based on previously conducted studies and does not contain any new studies with human participants or animals performed by any of the authors.

## Authors’ contributions

JF: Design, data generation, literature search, discussion, first draft, critical comments, final approval. AF, CS, FS: Literature search, discussion, final approval.

## Funding

No funding was received.

## Declaration of competing interest

The author declares that the research was conducted in the absence of any commercial or financial relationships that could be construed as a potential conflict of interest.
